# Radiotherapy volume delineation based on MRI and ^18^F-FDG-PET/MRI in locally recurrent rectal cancer

**DOI:** 10.1007/s00261-025-04859-2

**Published:** 2025-03-17

**Authors:** Yu-Kun Lin, Lei- Lei Zhu, Jun Zhao, Zuo-Lin Xiang

**Affiliations:** 1 Department of Radiation Oncology, Shanghai East Hospital, Tongji University School of Medicine, 150 Jimo Road, Pudong New District, Shanghai, China; 2 Department of Nuclear Medicine, Shanghai East Hospital, Tongji University School of Medicine, 150 Jimo Road, Pudong New District, Shanghai, China

**Keywords:** Radiotherapy, PET/MRI, Rectal cancer, MRI

## Abstract

**Objective:**

To evaluate the value of ^18^F-FDG-positron emission tomography (PET)/magnetic resonance imaging (MRI) functional imaging in the radiotherapy of locally recurrent rectal cancer by comparing the target volume delineation based on PET/MRI and MRI.

**Materials and methods:**

Twenty-six patients who were diagnosed with locally recurrent rectal cancer were included in this study. Patients underwent PET/MRI, and the target volume was delineated independently by three radiation oncologists. The degree of overlap, spatial consistency, and difference in the target volume delineated based on the two methods were compared. The efficacy of PET/MRI and MRI in detecting metastatic lymph nodes was analyzed.

**Results:**

In radiotherapy for patients with recurrent rectal cancer, the gross tumor volume (GTV), clinical target area (CTV), and nodal gross tumor volume (GTVn) delineated based on MRI and PET/MRI were correlated (*P* < 0.001, *P* < 0.001, and *P* < 0.001, respectively). Differences in CTV were statistically significant (*P* < 0.001), and the CTV greatly overlapped spatially. There is spatial heterogeneity in GTV and GTVn based on the two imaging modalities. Metastatic lymph node analysis revealed that the detection efficiency of the two modalities was the same at the population level. There was no significant difference in the number of metastatic lymph nodes detected (*P* = 0.521).

**Conclusion:**

PET/MRI can improve the accuracy of target volume delineation and has similar advantages to MRI in assessing the number of metastatic lymph nodes in patients with recurrent rectal cancer.

**Graphical abstract:**

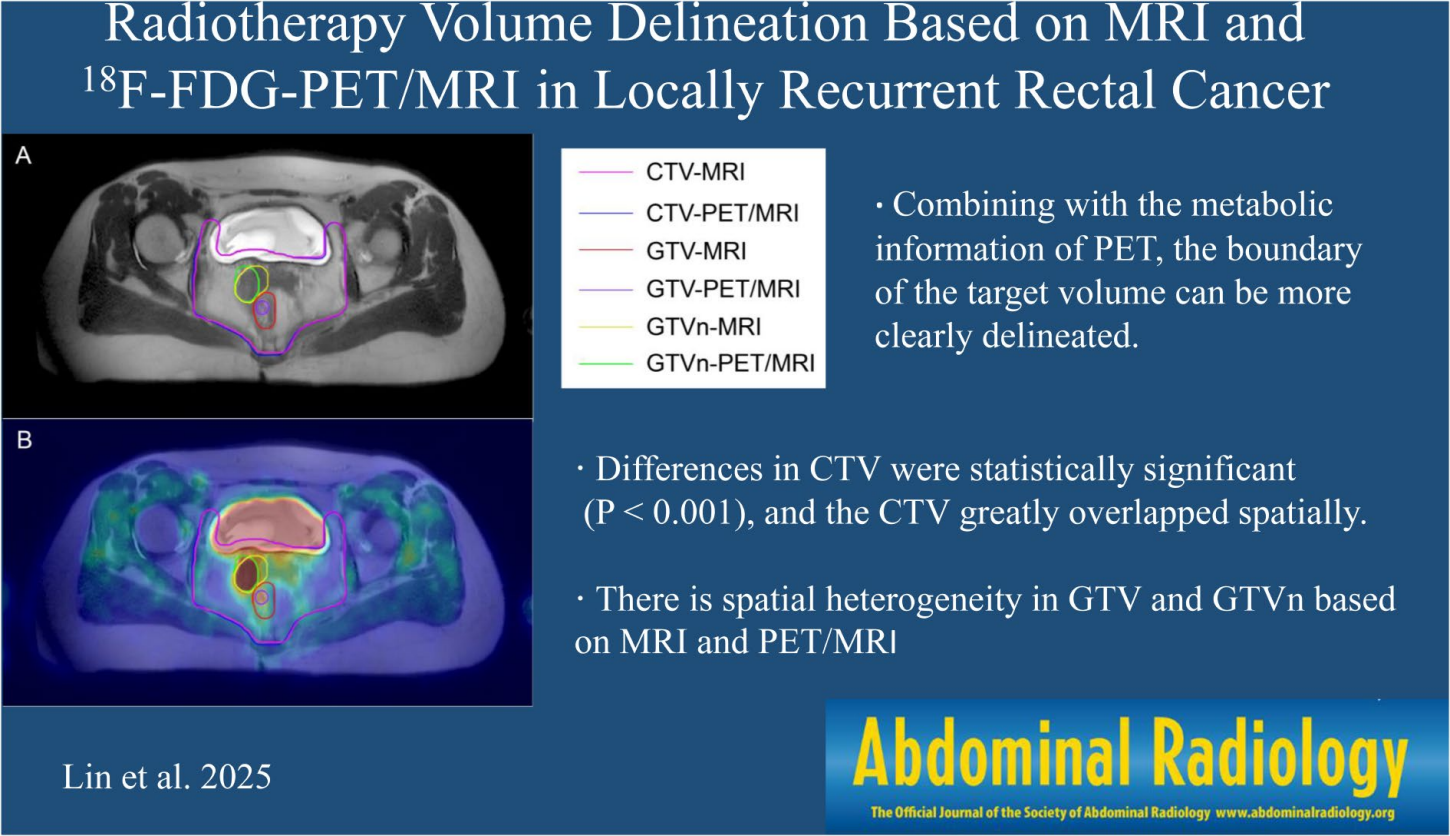

## Introduction

Colorectal cancer is the third most common cancer in the world [1], and its incidence is gradually increasing [2–5], making it a major public health problem [6]. In recent years, local recurrence and permanent colostomy have been greatly reduced [7, 8], with advances in tumor biology and staging, anatomical resection of the rectum, and the implementation of adjuvant and neoadjuvant therapy. The management of rectal cancer has improved in terms of locoregional failure (from 30 to 40% to less than 15%), postoperative mortality (from 10 to 2%), and conservative surgery (from 20 to 60%). The 5-year survival rate increased by 60%, reaching the level of colorectal cancer survival [9, 10].

Up to 40% patients with rectal cancer still experience either local or distant recurrence [11], and the risk of local recurrence ranges from 4–8% [12]. Patients with locally recurrent cancer present with complex symptoms, including pain, bleeding, fistulas, and malodorous discharge. Treatment for this condition is difficult. The prognosis in palliative care is dismal, with a 5-year survival rate of 5% [13]. However, neoadjuvant chemoradiotherapy and surgery can markedly improve patient prognosis, with a reported 5-year survival rate of more than 35% [14–18]. Locally recurrent rectal cancer can lead to a poor prognosis, including disabling pain or obstruction [7]. Thus, local recurrent rectal cancer remains an important clinical problem associated with increased morbidity, reduced reoperation success rates, and poor long-term survival.

A systemic treatment approach is needed for patients with local recurrence. Studies have shown that patients with untreated recurrent rectal cancer have a median survival of 3–8 months [19]. If radiotherapy and/or chemotherapy are involved, the median survival can be improved to 12–15 months [19, 20]. When these therapies are combined with radical surgery, the 5-year overall survival rate ranges from 25–36% [7]. Compared to those receiving surgical resection alone, patients treated with full or repeated radiotherapy before surgery have a lower rate of local recurrence [21]. Surgeons’ resection ability significantly improves with preoperative chemoradiotherapy. The percentage of patients with resectable disease increased from 29.2–64.9% [21], possibly due to reduction in the disease stage [22]. Reports indicate that performing surgery 6–8 weeks after radiotherapy can enhance the tumor response [23]. Palliative treatment is considered for symptomatic patients who are not suitable for curative multimodality therapy. Methods to improve quality of life and relieve symptoms include radiotherapy, local minimally invasive treatment, and surgery. Radiotherapy has been shown to improve bleeding and pain in 75% of patients [24]. Therefore, surgical resection is the main treatment for locally recurrent rectal cancer, and chemoradiotherapy is a necessary step to achieve a cure and improve resectability.

In the radiotherapy of rectal cancer, the efficacy largely depends on accurate delineation of the target volume, thereby allowing accurate anatomical adjustment of the dose delivery and target volume and maximizing the protection of the surrounding normal tissues, which depends on accurate imaging evaluation [25–27]. This can be a challenge because the recurrent patient has previously undergone primary site surgery and radiotherapy. Some patients experience chronic fistulas or anastomotic leakage, leading to a diagnosis of local recurrence due to extensive inflammation, post-treatment changes, and pelvic scarring [11]. Computed tomography (CT) and magnetic resonance imaging (MRI) are the two main imaging methods used to evaluate local cancer recurrence. Researchers report a sensitivity of 70% and a specificity of 85% for CT scanning of the lower abdomen in patients with locally recurrent rectal cancer. However, CT scanning has limitations, such as the inability to distinguish partial scars from tumor tissue [28]. In such cases, fluorine-18 2-fluoro-2-deoxy-D-glucose (^18^F-FDG) PET/CT can help distinguish post-treatment changes from recurrent tumors. It has been reported that PET/CT has a sensitivity of 98% and a specificity of 96% in differentiating benign from malignant ^18^F-FDG uptake. Thus, it generally has advantages over CT in detecting local recurrence [18]. MRI offers superior sensitivity because of its ability to distinguish soft tissue contrast resolution using different signal intensities. This makes the identification of normal tissue, scar tissue, and tumors different [7]. PET/MRI combines the metabolic activity information of PET and the high soft tissue contrast of MRI and has achieved high accuracy in the diagnosis of recurrent rectal cancer patients who have undergone primary resection [11].

Therefore, we propose that PET/MRI may improve the accuracy in radiotherapy. The value of PET/MRI in radiotherapy for locally recurrent rectal cancer was assessed by comparing the target volume delineated using MRI and ^18^F-FDG-PET/MRI.

## Materials and methods

### Patient selection

This study was approved by our ethical review committee. From August 2020 to September 2022, we retrospectively recruited patients who were admitted to Shanghai East Hospital with recurrent rectal cancer confirmed by needle biopsy, surgery, or colonoscopic pathological biopsy. The inclusion criteria were as follows: aged older than 18 years; underwent PET/MRI at our hospital; received surgery at the primary site before the PET/MRI scan; no history of other tumors or primary tumors at other sites; and no distant metastasis. Patients with incomplete images and excessive image motion artefacts were excluded. Twenty-six patients were ultimately included in this study. We collected demographic and clinical information from each patient, including sex, age, TNM stage, carcinoembryonic antigen (CEA) level, carbohydrate antigen 199 (CA199) level during the current PET/MRI examination, and pathological type.

### PET/MRI image acquisition

All patients underwent PET/MRI on a 3-Tesla PET/MR scanner (uPMR790 TOF, United Imaging, China). The patients fasted for more than 6 h before the examination, and after fasting blood glucose was less than 11.1 mmol/L, ^18^F-FDG was injected according to the standard of 0.10 mCi/kg. Whole-body PET scans were performed, covering five beds, with acquisition times of 3 min for each bed and 15 min for the pelvic bed. Image reconstruction was performed using the ordered subset expectation maximization (OSEM) algorithm (2 iterations, 20 subsets). Tissue segmentation was used for attenuation correction, and images were acquired with a 2-point Dioxon sequence and segmented for attenuation correction. The MRI scanning portion consisted of T1-weighted (T1W) high-resolution isotropic volume acquisition and T2-weighted (T2W) 3D volumetric fast spin‒echo imaging (oblique axial, coronal, and sagittal). Each patient’s MRI and PET/MRI images were reviewed visually, and written reports were generated by one board-certified radiologist and one nuclear medicine physician, both with more than 5 years of experience.

### Target volume delineation

Patient progress notes and imaging could be viewed through the Hospital Information System (HIS) and the Radiology Information System (RIS), and reports from radiologists and nuclear medicine physicians were provided. The images were uploaded to an Eclipse TPS workstation, and the target volume was independently delineated by three radiation oncologists.

Three radiation oncologists independently and manually delineated the target area on PET/MRI and MRI fusion images, including the gross tumor volume (GTV), clinical target volume (CTV), and nodal gross tumor volume (GTVn), based on T2-weighted MRI images. GTV includes the primary rectal tumor as identified on imaging. GTVn is delineated for patients with lymph node metastasis. The increased local uptake of lymph nodes on PET/MRI was considered to indicate metastasis, and a short-axis diameter of 1.0 cm for oval lymph nodes and a threshold of 0.8 cm for round lymph nodes were regarded as criteria for metastatic lymph nodes on MRI [29]. CTV includes the mesorectal area, presacral area, and internal iliac vascular area (pelvic lateral wall area), while the obturator area, external iliac area, anal complex area, and ischiorectal fossa may be irradiated according to the target volume delineation recommendations proposed by Valentini [30]. None of the three observers were aware of the patient’s past medical history or had seen any of the patients’ imaging reports before delineating the target volume.

### Statistical analysis

The GTV, CTV, and GTVn were delineated based on MRI and PET/MRI images. The conformity index (CI), lesion coverage factor (LCF), and Dice similarity coefficient (DSC) were used to compare the correspondence of target volumes. A and B represent the volumes depicted on MRI and PET/MRI, respectively (the same below). The CI was used to determine the similarity of two partial volumes, defined as A/B. The closer the CI results are to 1, the more similar the two parts are. LCF was used to determine the percentage overlap between the two parts, defined as (A∩B)/B. The closer the LCF results are to 1, the greater the overlap between the two volumes. DSC is used to determine the similarity of two parts in terms of volume and spatial consistency, defined as 2 × (A∩B)/(A + B). The DSC value equals 1 when two tumor lesions overlap completely; accordingly, when there is no overlap between them, the DSC value equals 0. It is generally accepted that the overlap is better when the DSC is greater than 0.7 [16, 31]. Bland‒Altman analysis was used to assess the consistency of the volumes delineated by the two methods.

**Table 1:** Demographic information for patients with recurrent rectal cancer

Characteristic	Value
**Age**	
Median (years)	59
**Sex** (%)	
Female	15 (57.7)
Male	11 (42.3)
**T Stage** (%)	
T0	3 (11.6)
T1	1 (3.8)
T2	2 (7.7)
T3	6 (23.1)
T4	14 (53.8)
**N Stage** (%)	
N0	5 (19.3)
N1	14 (53.8)
N2	7 (26.9)
**CEA**	
Mean (ng/mL)	8.046
**CA199**	
Mean (U/mL)	70.372
**Pathology** (%)	
adenocarcinoma	24 (92.3)
neuroendocrine neoplasm	2 (7.7)
**Radiotherapy** (%)	
Yes	13 (50.0)
No	13 (50.0)
**Chemotherapy** (%)	
Yes	21 (80.8)
No	5 (19.2)

Statistical analysis was performed using SPSS 26.0 (IBM Corp, Armonk, NY, USA) and R 4.2.1 (https://www.r-project.org/). The Wilcoxon signed rank test was used to compare the differences in volume delineated by the two methods. The linear mixed-effects model was applied to evaluate the differences in target volumes between MRI and PET/MRI while adjusting for potential confounding variables. Pearson analysis was used to evaluate the correlation, and the results are presented as a scatter plot. The difference in the detection efficacy for metastatic lymph nodes was compared by the Wilcoxon signed rank test. *P* < 0.05 was considered indicative of statistical significance.

## Results

### Patient characteristics

A total of 26 patients were recruited. The median age was 59 years; 57.7% were male, and 42.3% were female. According to postoperative pathology, 23.1% of the patients were in the T3 stage, 53.8% were in the T4 stage, and 80.7% had lymph node metastasis. The average value of CEA during the PET/MRI examination was 8.046 ng/mL, and the average value of CA199 was 70.372 U/mL. The clinical and demographic information is shown in Table 1. All patients underwent surgery after the initial diagnosis, including 18 patients who underwent low anterior resection (LAR) and 4 patients who underwent abdominoperineal resection of the rectum (Miles); two patients underwent Hartmann’s procedure, and two patients underwent endoscopic therapy. Pathology revealed that 24 (92.3%) patients had adenocarcinoma, and 2 (7.7%) patients had neuroendocrine tumors. 50% of patients received radiotherapy during the course of the disease, and 80.8% received chemotherapy.

**Table 2:** Gross tumor volume and related parameters

Characteristic (*n* = 26)	MRI volume (cm^3^)	PET/MRI volume (cm^3^)	Overlap Volume (cm^3^)	CI	LSF	DSC
Mean	26.3	20.0	14.6	1.590	0.785	0.670
SD	25.3	20.7	15.8	0.877	0.258	0.176

CI, conformity index; LCF, lesion coverage factor; DSC, Dice similarity coefficient

**Table 3:** Clinical tumor volume and related parameters

Characteristic (*n* = 26)	MRI volume (cm^3^)	PET/MRI volume (cm^3^)	Overlap Volume (cm^3^)	CI	LSF	DSC
Mean	755.4	730.6	700.0	1.034	0.958	0.941
SD	117.5	114.5	112.8	0.026	0.019	0.016

CI, conformity index; LCF, lesion coverage factor; DSC, Dice similarity coefficient

**Table 4:** Nodal gross tumor volume and related parameters

Characteristic (*n* = 26)	MRI volume (cm^3^)	PET/MRI volume (cm^3^)	Overlap Volume (cm^3^)	CI	LSF	DSC
Mean	13.6	12.3	8.8	2.326	0.679	0.557
SD	13.6	14.0	10.7	3.916	0.247	0.226

CI, conformity index; LCF, lesion coverage factor; DSC, Dice similarity coefficient

**Fig. 1:**
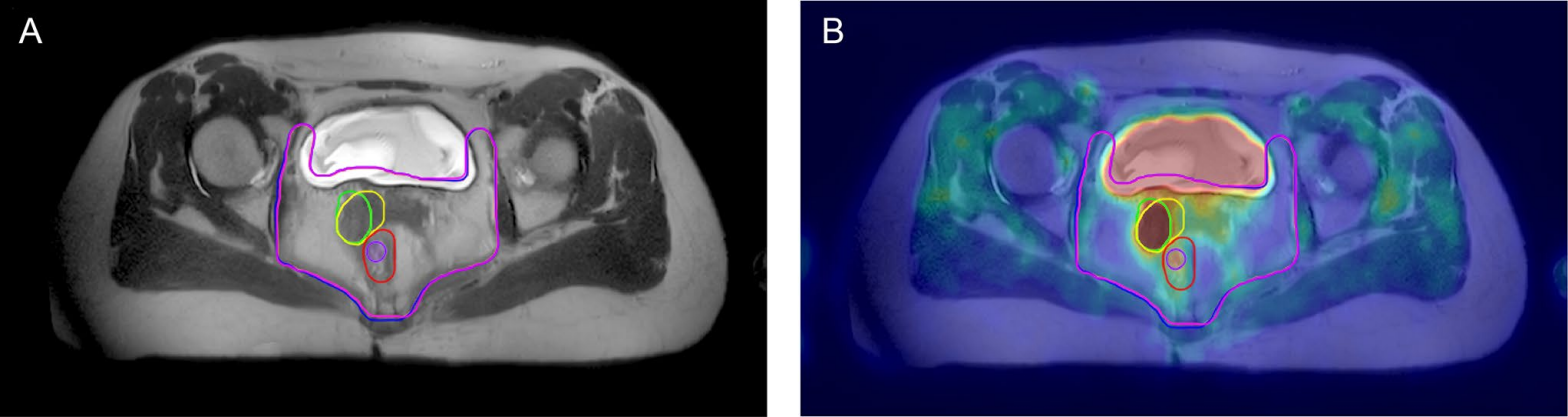
GTV and GTVn delineated for a 38 years old female in red and yellow lines based on MRI (**A**); GTV and GTVn delineated in purple and green lines based on PET/MRI (**B**); CTV delineated based on MRI and PET/MRI are represented by pink and blue lines, respectively. Combining with the metabolic information of PET, the boundary of the target volume can be more clearly delineated

**Fig. 2:**
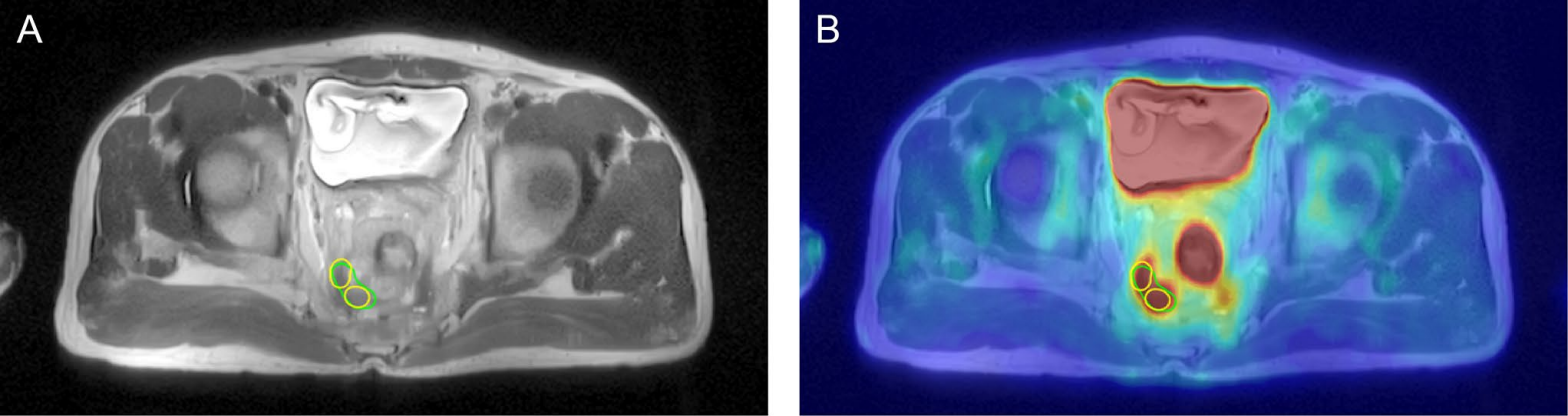
GTVn delineated for a 52 years old male on MRI (**A**) and PET/MRI (**B**). MRI image (**A**) delineated two smaller target volumes (yellow line) due to blurred boundaries, and PET/MRI image (**B**) showed complete GTVn (green line)

### Volume measurements

**Fig. 3:**
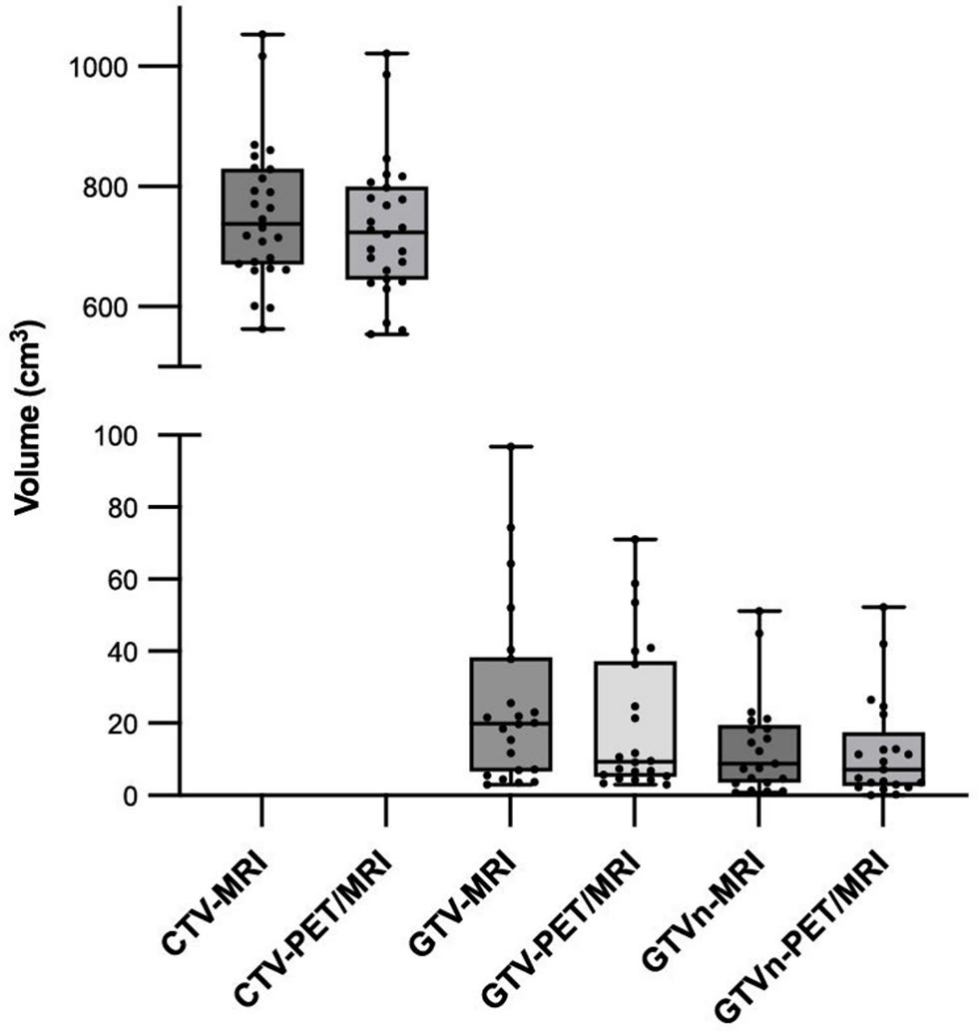
Distribution of the target volume based on MRI and PET/MRI

Radiation oncologists used the Eclipse TPS workstation to delineate the CTV, GTV, and GTVn, as shown in Figs. 1–2. The average values and distribution of the relevant parameters of the target volume are shown in Tables 2, 3 and 4; Fig. 3.

**Fig. 4:**
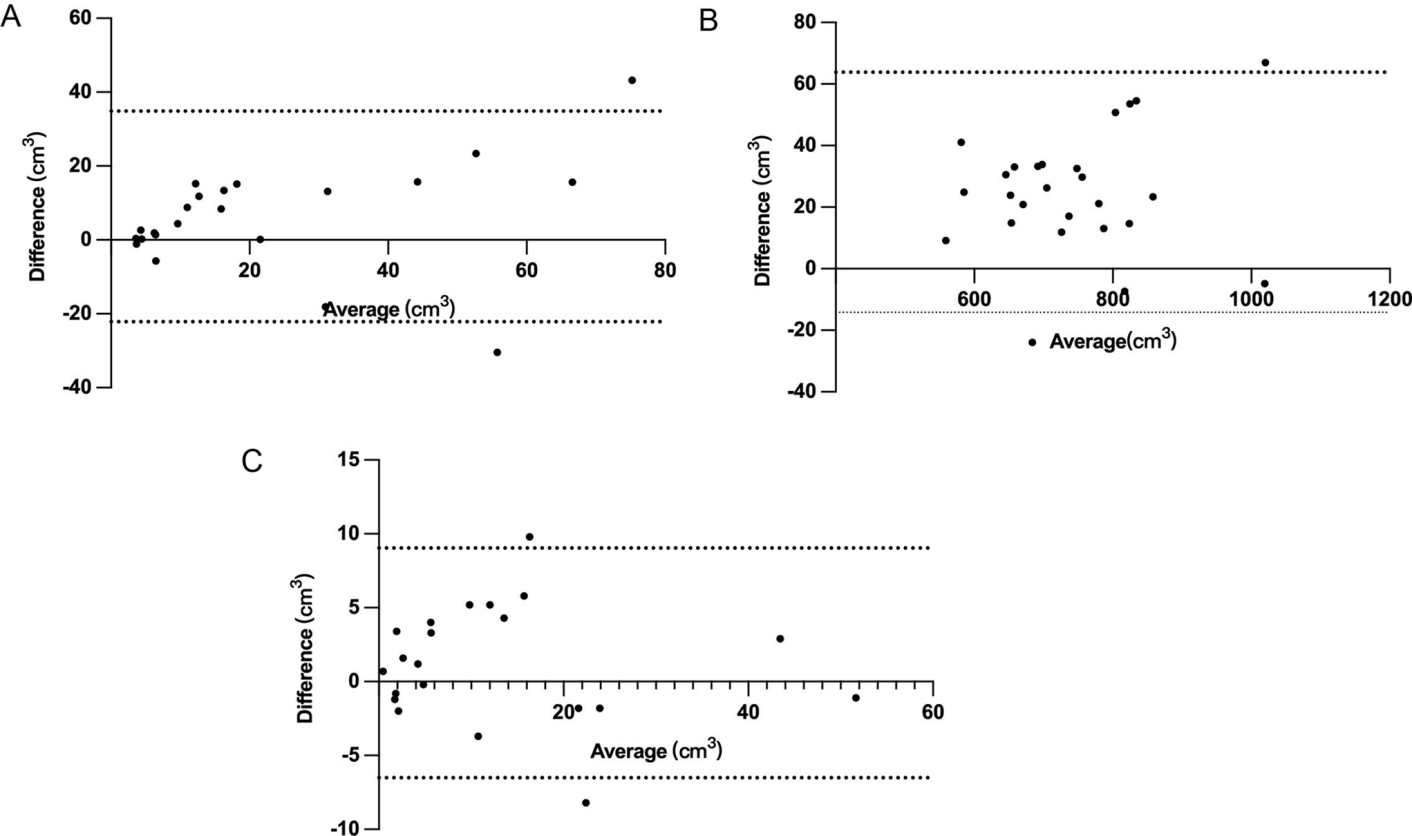
Bland-Altman analysis of GTV (**A**), CTV (**B**) and GTVn (**C**) between the two modalities. The area between the dotted lines represents the 95% confidence intervals

For GTV delineation, the average volume based on MRI was 26.3 cm^3^, and the average volume based on PET/MRI was 20.0 cm^3^. The CI, LCF, and DSC were 1.590 ± 0.877, 0.785 ± 0.258, and 0.670 ± 0.176, respectively. For CTV delineation, the average volume based on MRI was 755.4 cm^3^, and the average volume based on PET/MRI was 730.6 cm^3^. The CI, LCF, and DSC were 1.034 ± 0.026, 0.958 ± 0.019, and 0.941 ± 0.016, respectively. In terms of metastatic lymph node regions, the mean GTVn based on PET/MRI was slightly smaller compared with MRI. The mean CI, LCF, and DSC were 2.326 ± 3.916, 0.679 ± 0.247, and 0.557 ± 0.226, respectively. According to the DSC values, the CTV obtained based on the two methods exhibited good spatial overlap.

Bland-Altman analysis revealed that the mean differences in GTV, CTV, and GTVn were 6.332, 24.796, and 1.267, with 95% confidence intervals ranging from − 0.117 to 12.781, 16.757 to 32.835, and − 0.539 to 3.072, respectively, as shown in Fig. 4. For GTV, 90.9% of the measured volumes were within the 95% CI. For CTV, 92.3% of the measured values were within the 95% CI. For GTVn, 90.5% of the measured values were within the 95% CI, indicating that the volume measured by the two methods showed good consistency.

Correlation analysis revealed that the GTV (*r* = 0.818, *P* < 0.001), CTV (*r* = 0.986, *P* < 0.001), and GTVn (*r* = 0.959, *P* < 0.001) delineated by the two methods were related. The Wilcoxon signed rank test indicated that there were significant differences in GTV and CTV based on MRI and PET/MRI images (*P* = 0.017, *P* < 0.001), but there was no significant difference in GTVn volume (*P* = 0.154). The linear mixed-effects model was further used to evaluate the effect of different imaging modalities on CTV and GTV, adjusting for covariates including age, gender, T stage, N stage, radiotherapy, and chemotherapy. The results showed that there was a significant difference in CTV volume delineated by different imaging methods (*P* < 0.001), while no significant difference was observed in GTV volume (*P* = 0.054).

### Metastatic lymph node detection

Among the 26 patients included in the study, metastatic lymph node analysis by imaging was performed. In total, 21 patients had lymph node metastasis (LNM) identified using both MRI and PET/MRI. The average number of positive lymph nodes detected by PET/MRI was 4.050 ± 3.369, and that by MRI was 4.190 ± 3.487. There was no significant difference in the number of metastatic lymph nodes detected by the two methods (*P* = 0.521).

## Discussion

This study compared the radiotherapy target volume detected by PET/MRI and MRI in order to evaluate the value of PET/MRI functional imaging for target delineation in patients with postoperative recurrent rectal cancer. We found that the target volume differed between the two imaging methods, and there was spatial heterogeneity between them.

Studies have shown that after curative surgery for rectal cancer, local recurrence rates range from 2.4 to 10%, and distant metastasis rates range from 20 to 50% [1]. In recent years, improvements in surgical methods and the application of neoadjuvant therapy for rectal cancer have successfully reduced the postoperative local recurrence rate [12]. However, local recurrence is still a thorny problem affecting the prognosis of patients, and the management of the recurrence site requires a multidisciplinary expert team [7], with radiotherapy playing an essential role. Accurate evaluation and diagnosis of lesions by imaging can reduce the risk of treatment failure and prolong patient survival.

Currently, CT remains the standard imaging method for target volume delineation and conformal radiotherapy treatment plans. PET/CT has been used in some studies for radiotherapy planning in rectal cancer patients, and the use of PET/CT has been reported to benefit patients by helping to avoid geographic omissions and by facilitating more accurate adjustment of the caudal boundary of the lesion during tumor delineation [32]. A meta-analysis demonstrated that PET/CT and whole-body PET are valuable imaging tools for detecting and localizing colorectal cancer recurrence in the presence of elevated CEA levels [33]. In a study of 44 rectal cancer resection patients, the sensitivity of PET/MRI for detecting recurrence was 94%, and the specificity was 94% [11]. Plodeck et al. [34] assessed the efficacy of PET/MRI in the diagnosis of recurrent rectal cancer. MRI and PET/MRI are accurate in the diagnosis of recurrent tumors, and PET/MRI improves the confidence level of readers. The number of patients with equivocal MRI findings was reduced (5% vs. 12%). Therefore, we propose that PET/MRI can improve the accuracy of target volume delineation for locally recurrent rectal cancer patients. The GTV, CTV, and GTVn were delineated and compared based on hybrid PET/MRI and MRI images. Volume data showed that smaller target volumes could be obtained based on PET/MRI. Bland-Altman analysis and consistency analysis showed that the GTV, CTV, and GTVn delineated by the two methods had good consistency and did not change with the volume. Further difference analysis and multivariate analysis showed that CTV based on MRI and PET/MRI was significantly different. Spatial volume analysis showed a high degree of spatial overlap among CTVs. This indicates that CTV can be delineated more accurately based on PET/MRI. Although no statistically significant difference was observed in the target volumes of GTV and GTVn between the two imaging modalities, the DSC values were less than 0.7, suggesting spatial heterogeneity and poor overlap between the two regions. These differences may be due to the difficulty in differentiating benign changes from locally recurrent lesions on MRI. Locally recurrent tumor tissue typically has a higher signal than muscle on T2W MRI [35], however these high signal intensities may also correspond to granulation tissue, hematoma, and radiation-induced inflammatory changes [36]. At the same time, it is difficult to distinguish benign fibrosis caused by previous surgery and its complications or adjuvant therapy from local recurrent tissue [37], resulting in contouring errors in the target volume. PET/MRI combines functional imaging with excellent soft tissue contrast, making it very useful for patients with recurrent tumors [34]. It can help distinguish post-treatment scars, connective tissue hyperplasia reactions from residual tumors or local recurrence. A smaller target volume based on PET/MRI may produce less normal tissue radiation, reduce complications, and improve the quality of life of patients. Therefore, we believe that PET/MRI has advantages in accurately delineating the target volume of rectal radiotherapy. Several studies have shown that PET/MRI provides more information for radiotherapy planning. This finding is consistent with our results despite different tumor locations [38–40].

Our study showed that PET/MRI detected a slightly smaller number than MRI in evaluating suspected metastatic lymph nodes. The sensitivity of PET/MRI is influenced by the size of LNM. Research has shown that the sensitivity is 57.1% for LNMs larger than 5 mm, while it decreases to 0% for LNMs smaller than or equal to 5 mm [41]. Meanwhile, in cases of necrotic lymph nodes, the lack of sufficient tracer uptake by the lesion results in false-negative findings [42]. In the analysis of patients with LNM, the two methods had similar efficacy. We compared the number of positive lymph nodes detected by the two methods, and there was no significant difference between them. Catalano et al. [3] evaluated the lymph node accuracy of two assays at the lymph node level, with a sensitivity of 72% (95% CI, 0.66 to 0.77) for MRI. The sensitivity of PET/MRI was 82% (95% CI, 0.77–0.86). There was no significant difference in performance at the lymph node level (*P* = 0.82), similar to our results. Based on the above results, we believe that PET/MRI has similar advantages to MRI in evaluating positive lymph nodes in patients with recurrent rectal cancer, such as good tissue resolution. The dedicated MRI component of PET/MRI has the potential to provide moderate accuracy in the N staging of rectal cancer, and the PET component can provide additional information about glucose metabolism, which can enhance the assessment of lymph node metastasis [43]. Because most of the patients included in the study did not undergo surgical treatment after recurrence, it was not possible to obtain pathological biopsy data or imaging data for metastatic lymph nodes. It is necessary to directly compare the sensitivity and specificity of MRI and PET/MRI for LNM detection with those of pathological results in large samples in the future.

The main limitation of this study is the relatively small number of patients recruited. Due to the short time in which PET/MRI has been in use at our center, the amount of large-scale research has been limited. Furthermore, due to the insufficient follow-up time and the inherent limitations of retrospective study, the prognosis of recurrent rectal cancer patients undergoing radiotherapy based on PET/MRI remains unclear. Therefore, there is still an urgent need for more prospective, multicenter, and large-sample studies to look at outcomes in patients undergoing PET/MRI compared with MRI alone.

## Conclusion

It is feasible to visually delineate the target volume for locally recurrent rectal cancer based on PET/MRI. In addition, PET/MRI possesses similar advantages to MRI in evaluating metastatic lymph nodes. PET/MRI has broad potential in the development of personalized and precise radiotherapy for locally recurrent rectal cancer patients.

## Data Availability

Data, analytical methods, and study materials will be made available to other researchers from the corresponding author upon reasonable request.

## Abbreviations

CT: computed tomography simulation
MRI: magnetic resonance imaging
^18^F-FDG: fluorine-18 2-fluoro-2-deoxy-D-glucose
CEA: carcinoembryonic antigen
CA199: carbohydrate antigen 199
OSEM: ordered subset expectation maximization
T1W: T1-weighted
T2W: T2-weighted
HIS: Hospital Information System
RIS: Radiology Information System
GTV: gross tumor volume
CTV: clinical target volume
GTVn: nodal gross tumor volume
CI: conformity index
LCF: lesion coverage factor
DSC: Dice similarity coefficient
LNM: lymph node metastasis

## Declarations

### Provenance and peer review

Not commissioned, externally peer-reviewed.
